# Supplementary figures and images for: Mapping genetic effects on cell type-specific chromatin accessibility and annotating complex immune trait variants using single nucleus ATAC-seq in peripheral blood
Source: PLoS Genet. 2023 Jun 8;19(6):e1010759. doi: 10.1371/journal.pgen.1010759 (PMC10298776; doi:10.1371/journal.pgen.1010759)

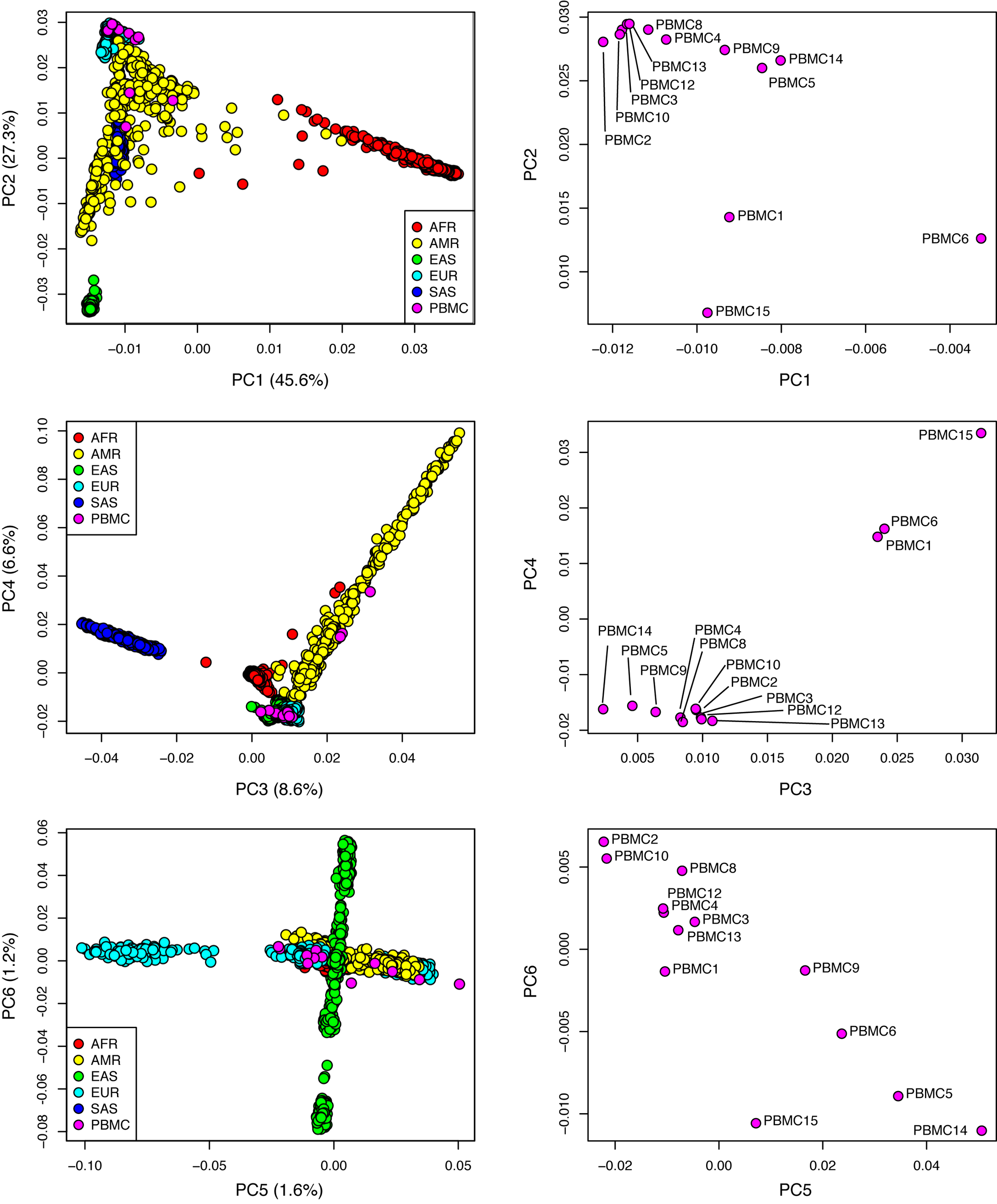

Supplement: S1 Fig — The first six principal components derived from joint analysis of genotype data from the 1000 Genomes Project and PBMC samples. Samples in 1000 Genomes are colored by major population group, and the PBMC samples are colored in pink, and further shown in separate plots on the right. (TIF) [file pgen.1010759.s001.tif]

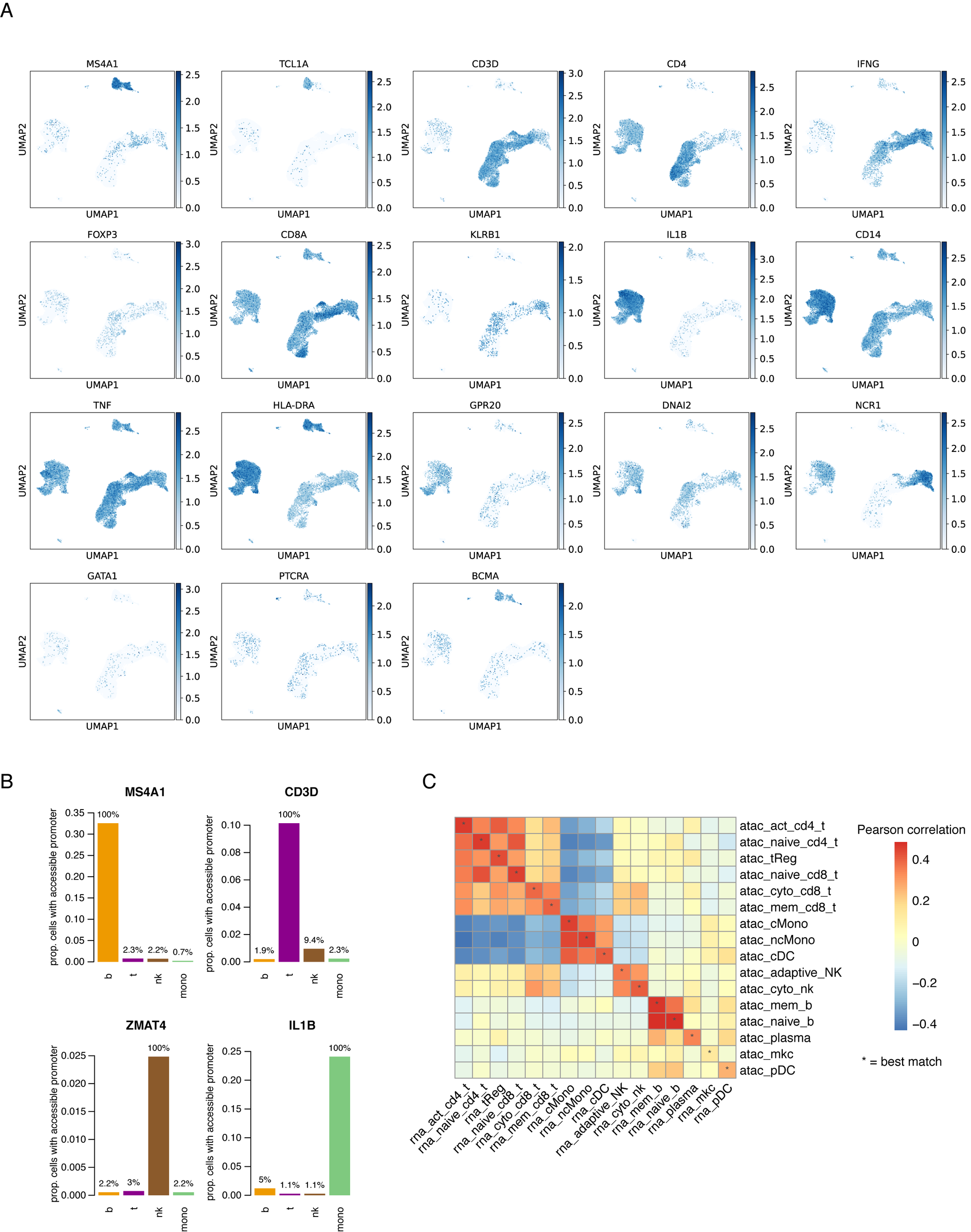

Supplement: S2 Fig — A) UMAP plots showing promoter accessibility in a 1 kb window around the TSS for selected cell type marker genes (see S3 Table). B) Proportion of nuclei in each cell type with at least one read mapping to a promoter peak for cell-type specific genes. The percentage of nuclei with accessible marker promoter with respect to the correct cell type is shown on top of each bar. C) Heatmap showing Pearson correlation between the top cluster-specific genes in scRNA-seq and snATAC-seq clusters using a Wilcoxon signed-rank test. (TIF) [file pgen.1010759.s002.tif]

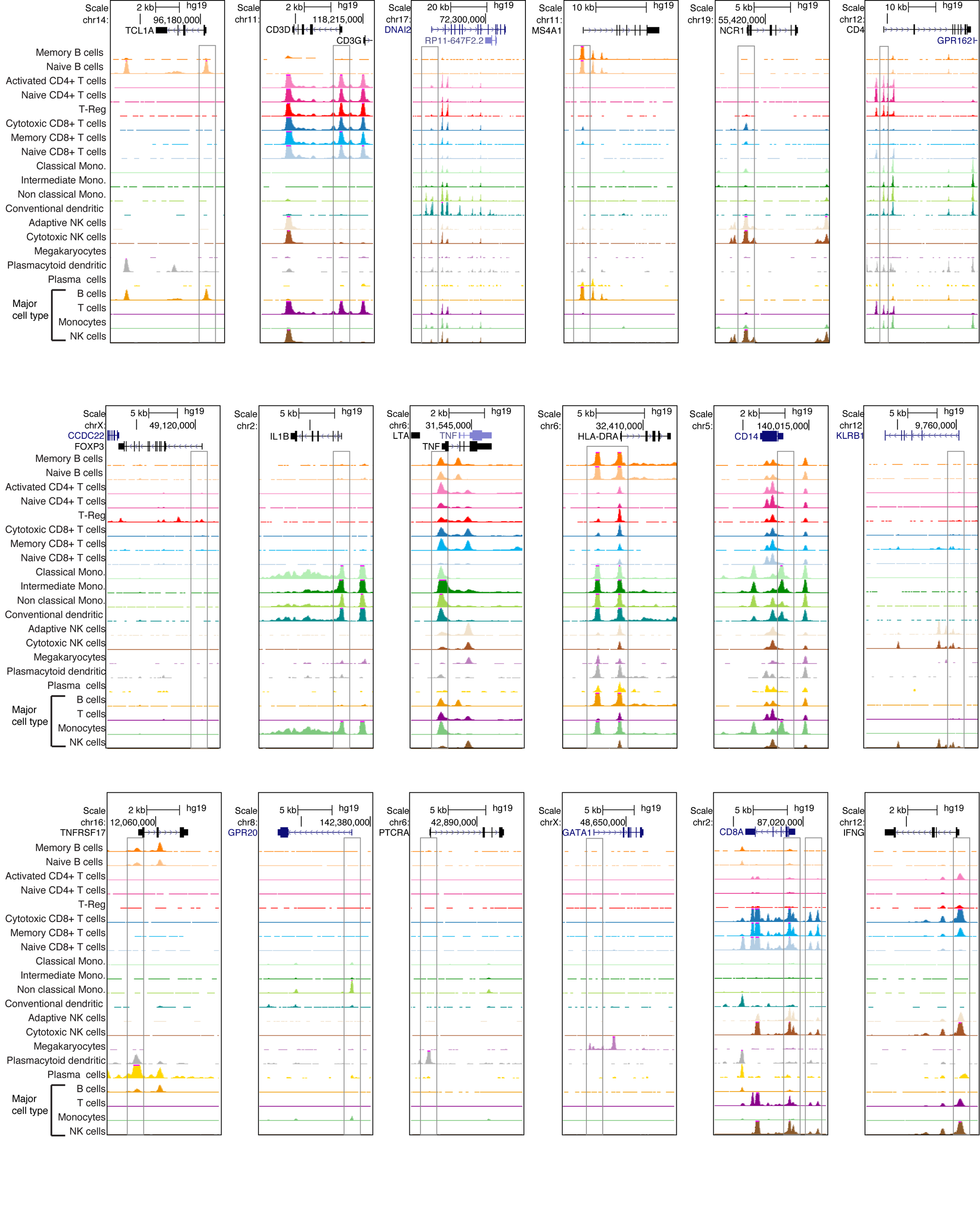

Supplement: S3 Fig — Genome browser plots showing aggregate read density (scaled to 1×10^5^ read depth, y-axis: 0–8 normalized read depth) for cells within each cell type for selected cell type marker genes. (TIF) [file pgen.1010759.s003.tif]

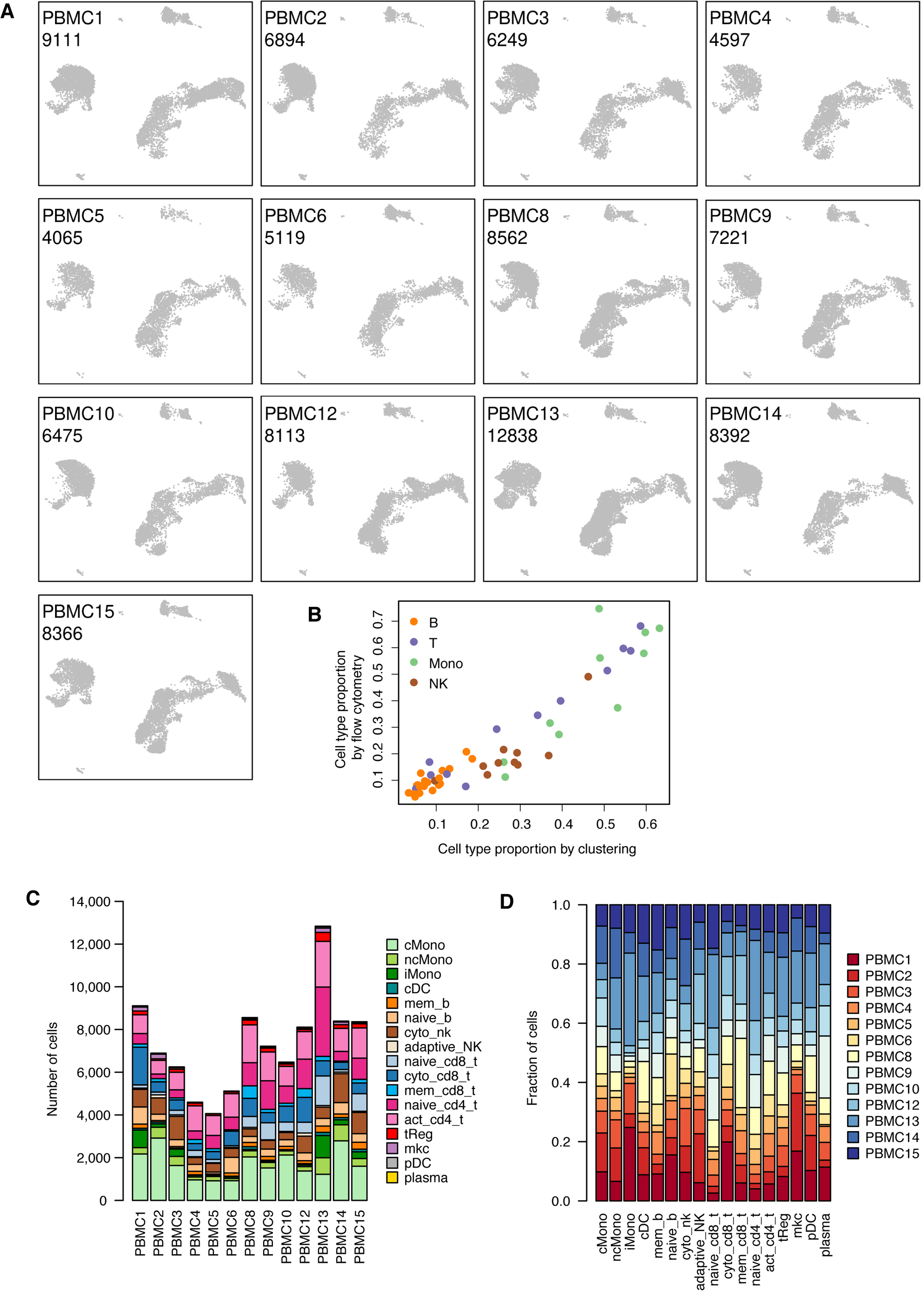

Supplement: S4 Fig — A) UMAP plot showing cells in each of the 13 PBMC samples assayed in this study. B) Scatter plot comparing cell type proportions obtained from cluster analysis versus those obtained from flow cytometry, excluding leukocytes. Proportions represent the fraction of all cells in each sample (see S4 Table for individual sample proportions). Each dot represents an individual sample. C) Barplot showing the number of cells assigned to 17 distinct immune cell types and sub-types in each sample. D) Barplot showing the relative proportion of cells from each sample in each immune cell type and sub-type. (TIF) [file pgen.1010759.s004.tif]

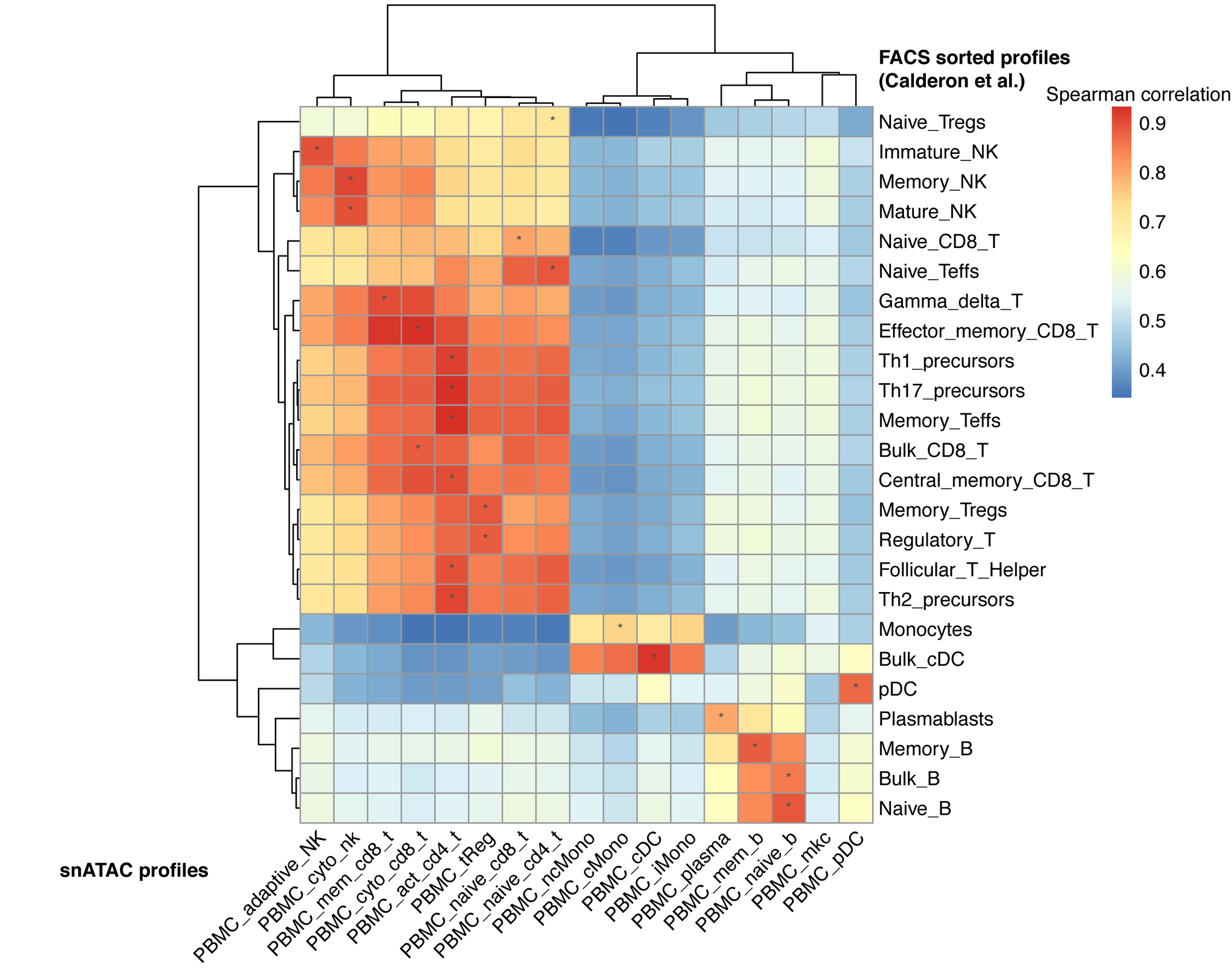

Supplement: S5 Fig — Heatmaps and hierarchical clustering of Spearman correlation coefficients for pairwise comparisons of genome-wide ATAC-seq profiles across cell sub-types from PBMC snATAC-seq from this study (columns) and from a published bulk ATAC-seq study of FACS sorted immune cells (rows). An asterisk denotes the best matching (highest correlation) cell type from snATAC-seq to FACS-sorted immune cells. (TIF) [file pgen.1010759.s005.tif]

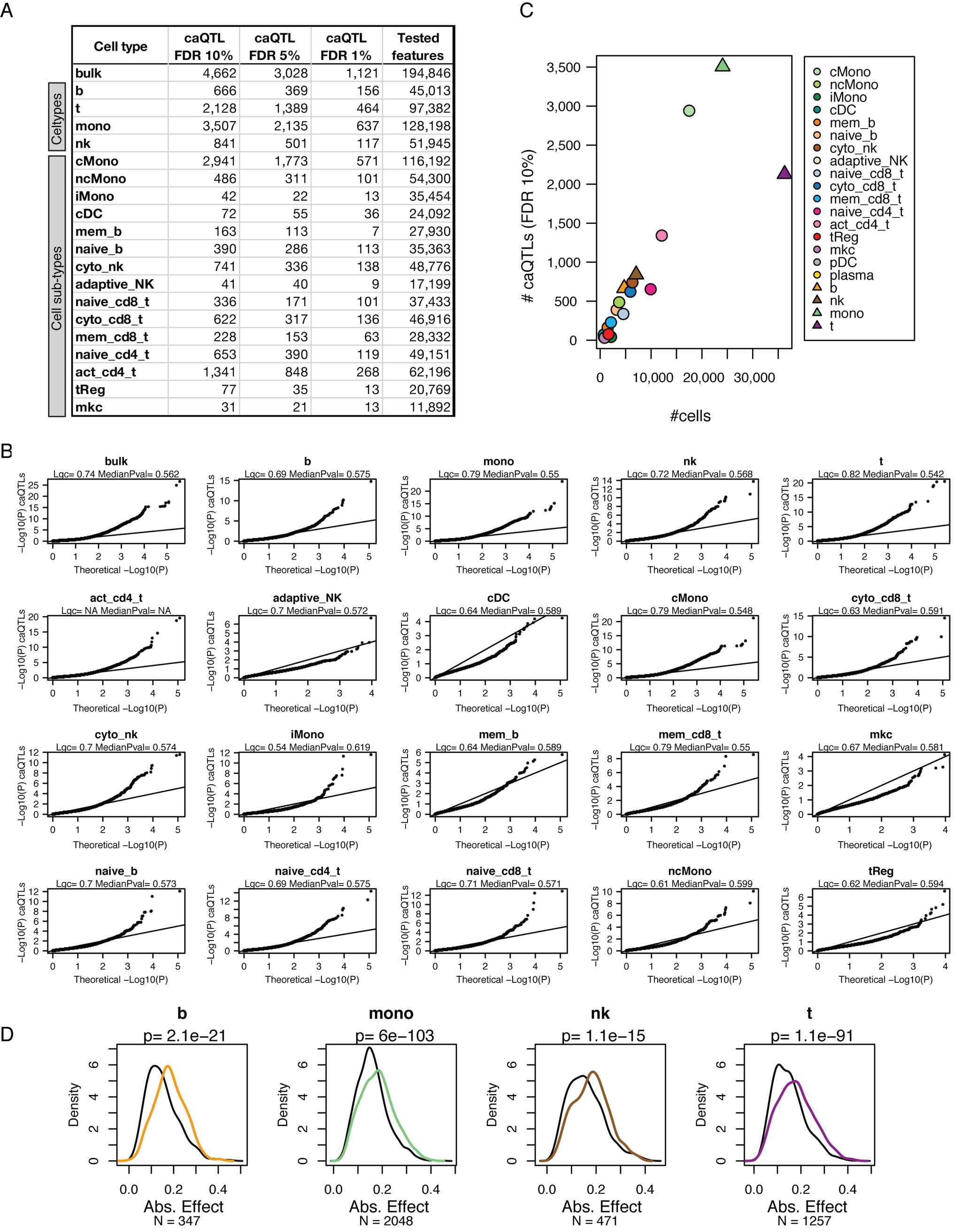

Supplement: S6 Fig — A) Number of caQTLs identified in each cell type, sub-type, or”bulk” resolution, at different FDR thresholds. B) Q-Q plots of nominal p-values (one top variant per peak) for each caQTL dataset. C) Scatterplot showing correlation between number of cells per cell type and number of caQTLs identified. D) Comparison of effect sizes (converted to effect allele direction) at shared sites between “bulk” (black) and major cell types (color) caQTLs. P-value of two-sided paired Wilcoxon test is shown. (TIF) [file pgen.1010759.s006.tif]

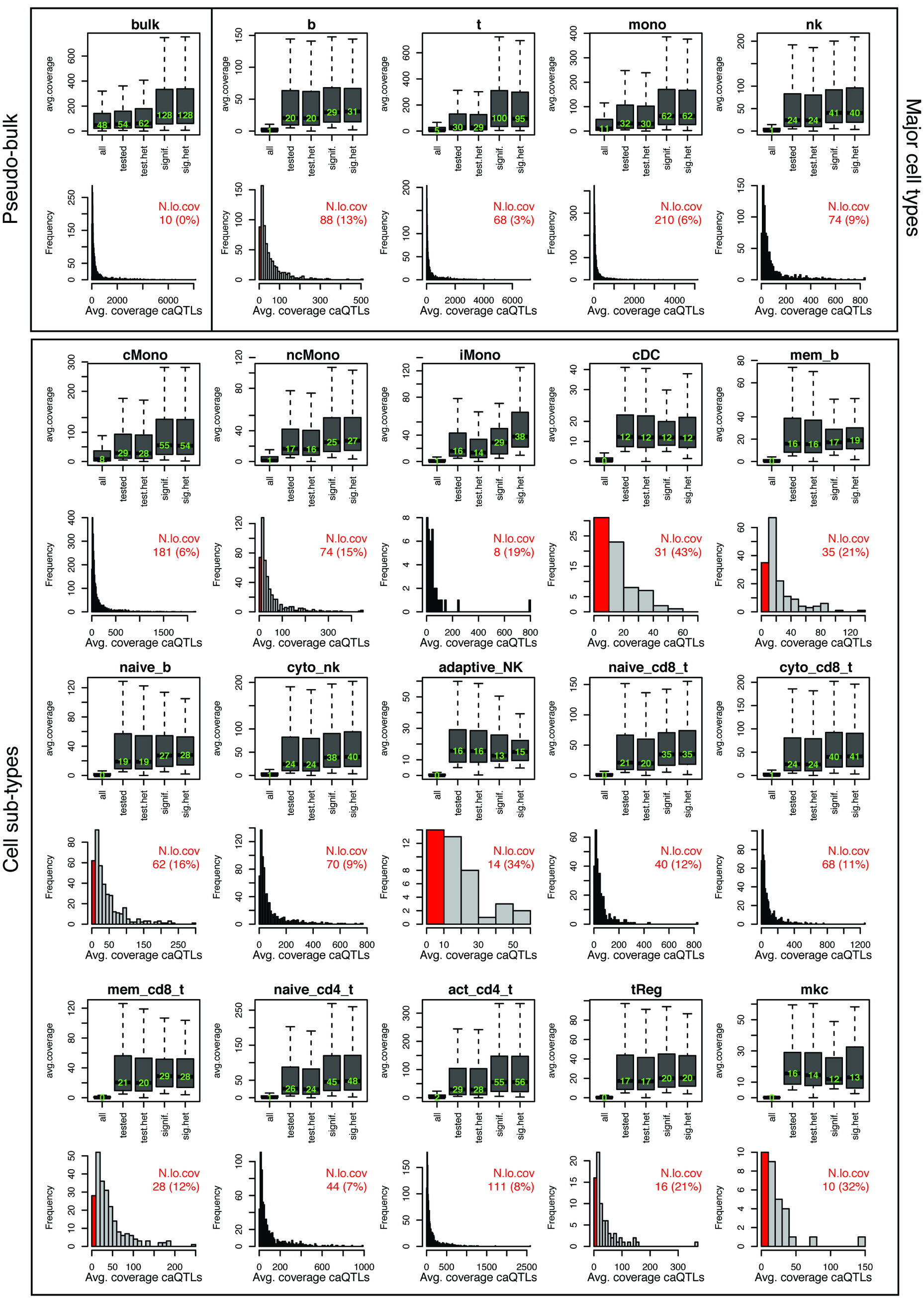

Supplement: S7 Fig — Boxplots for cell types, sub-types, and”bulk” show the distribution of mean read depth for all peaks, tested peaks, and significant caQTL peaks in either all samples or only heterozygous samples. The median of the distribution is indicated in green. The caQTLs (last 2 boxplots) have higher median coverage compared to tested peaks. The histograms on the bottom show the average read depth for caQTLs, with the number of peaks with lower coverage (mean <10 reads) shown in red. (TIF) [file pgen.1010759.s007.tif]

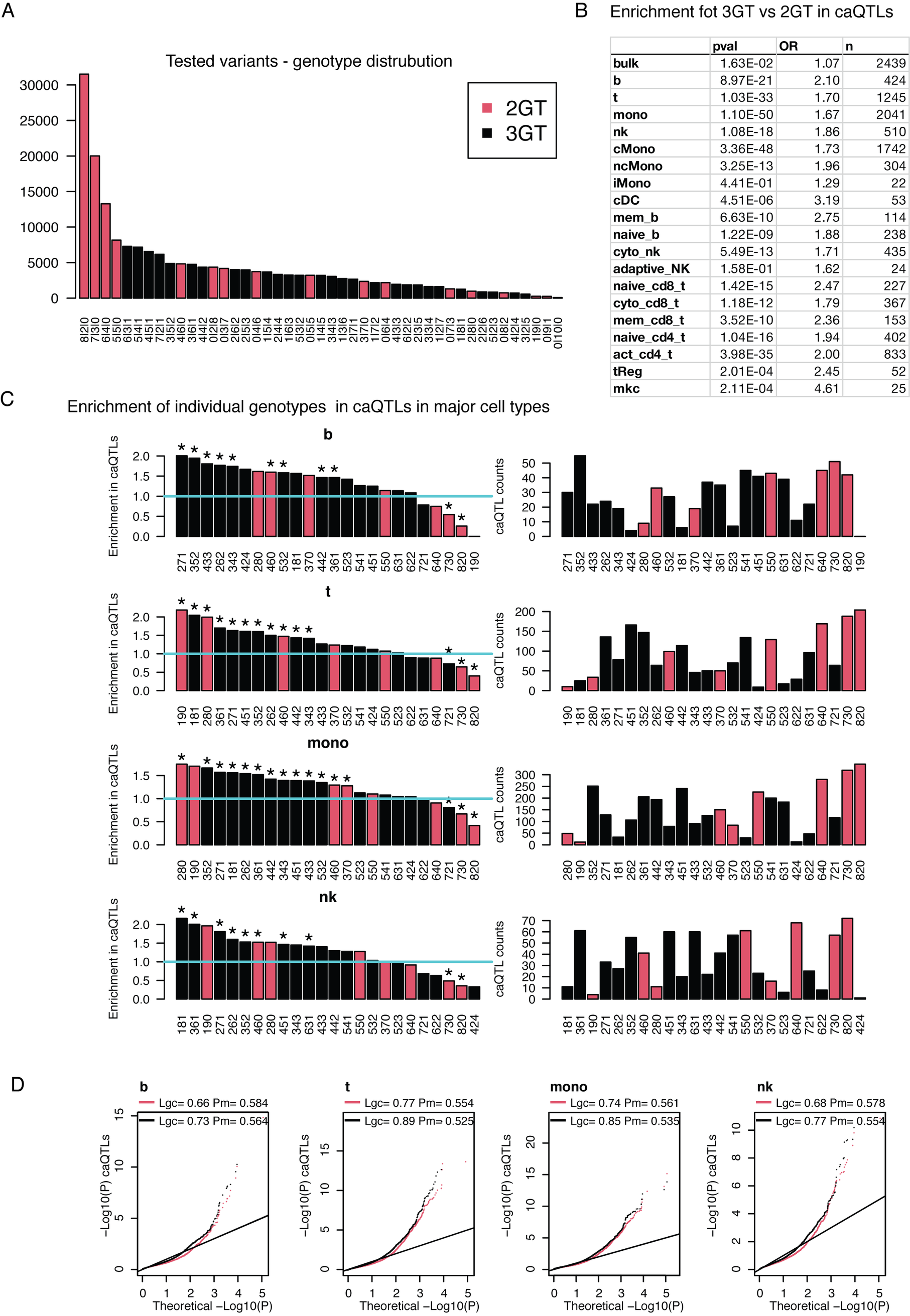

Supplement: S8 Fig — A) Distribution of genotype classes across all tested variants indicated as number of individuals with homozygous reference, heterozygous and homozygous alternate alleles. Two- and three-genotype classes are color-coded by red and black, respectively. B) Fisher’s exact test results showing enrichment for three-genotype classes in caQTLs in each cell type, sub-type, or “bulk”. C) Fisher’s exact test enrichment results for individual genotype classes in B cells, T cells, Monocytes and NK cells. D) Q-Q plots of lead SNPs stratified between two- (red) and three- (black) genotype classes. (TIF) [file pgen.1010759.s008.tif]

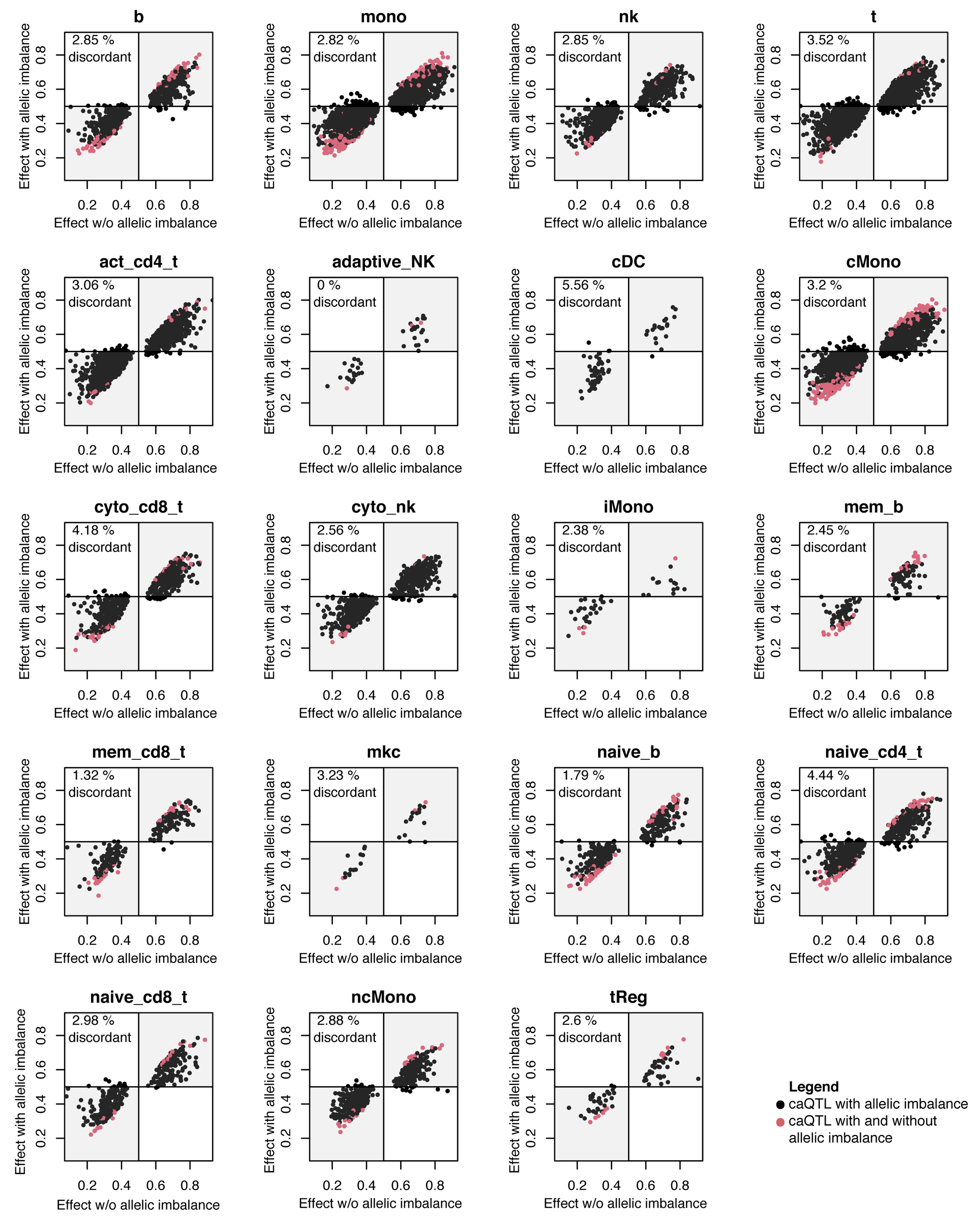

Supplement: S9 Fig — For each cell type (first 4 plots) and sub-type (remaining 15 plots), a scatter plot shows the consistency between caQTL effect (π) in the standard QTL analysis including allelic imbalance (x-axis,) and the effect for the same variant-peak pair excluding the allelic imbalance component (y-axis). The latter was obtained by running RASQUAL using the—population-only option. Black dots indicate caQTLs that were significant only in the standard analysis, and red dots indicate caQTLs that were significant in both analyses. The percentage of discordant effects are indicated. (TIF) [file pgen.1010759.s009.tif]

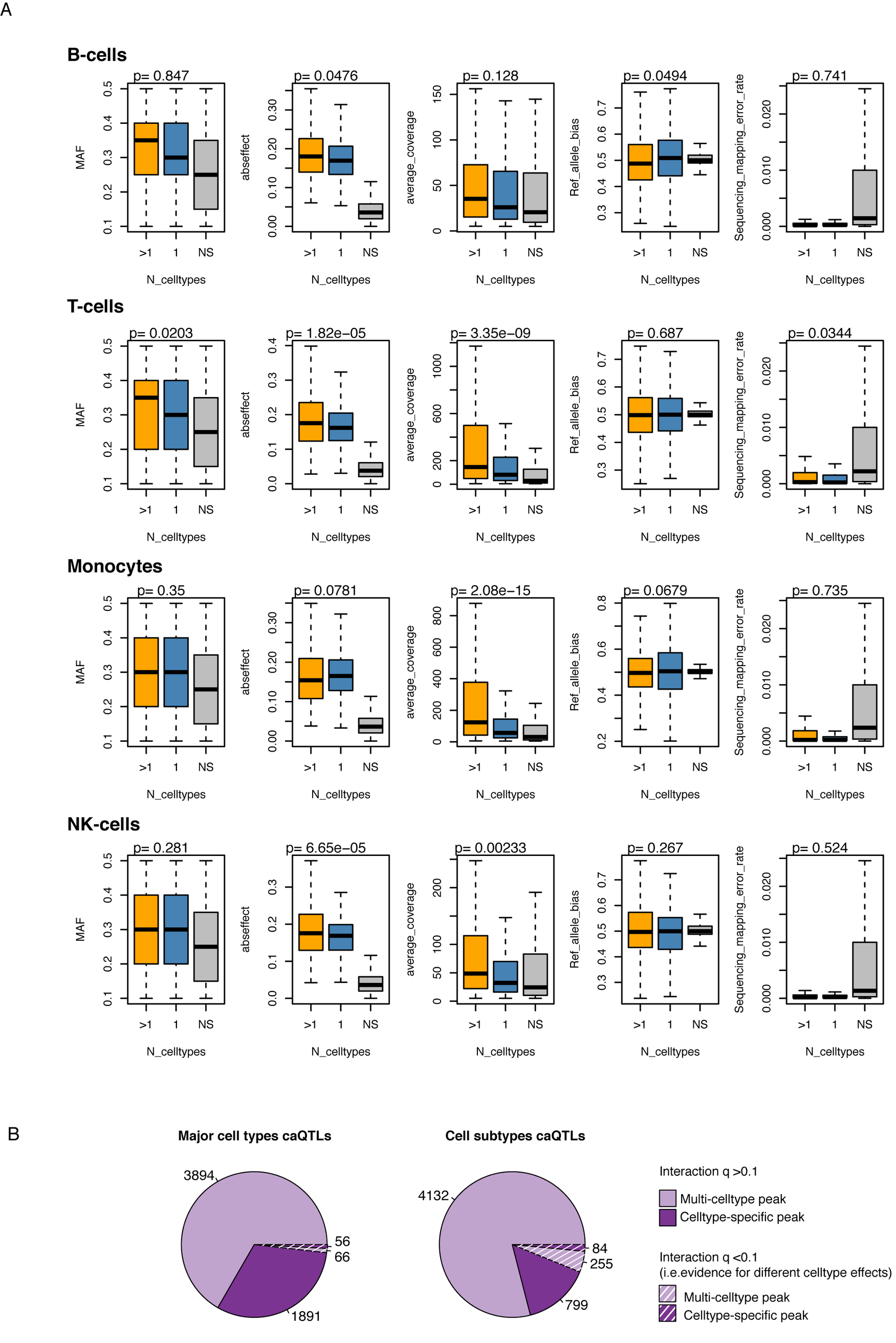

Supplement: S10 Fig — A) Properties of caQTLs shared by more than one cell type and present only in that cell type, as well as non-caQTLs (NS), for the four major cell types (B cells, T cells, Monocytes, NK cells). MAF = minor allele frequency within PBMC samples, abseffect = “absolute” (effect allele) effect size. P-values are from a two-tailed t-test. B) Number of caQTLs with cell-type specific effects based on ANOVA and classified by whether the caQTL is for a cell type-specific or cell type-shared peak. (TIF) [file pgen.1010759.s010.tif]

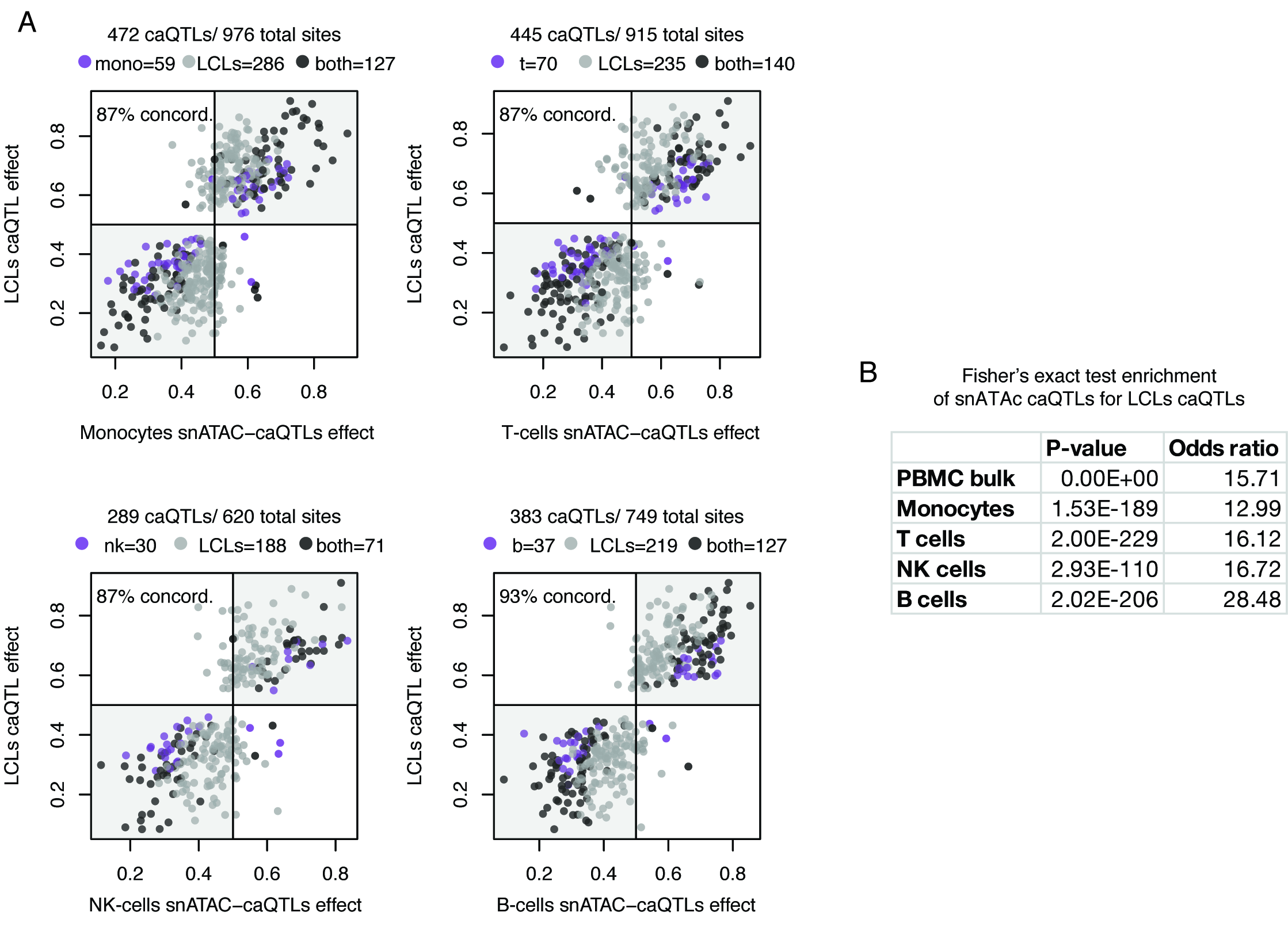

Supplement: S11 Fig — A) Significant caQTLs in Monocytes, T-cells, NK-cells and B-cells and their overlap with LCL caQTLs, considering only peaks tested in both datasets and where the same variant was tested. For caQTLs identified in either study (indicated in the legend), a scatter plot shows effect sizes for each study. B) P-values and odds ratio from a two-tailed Fisher’s exact test for enrichment of cell type caQTLs in LCL caQTLs. (TIF) [file pgen.1010759.s011.tif]

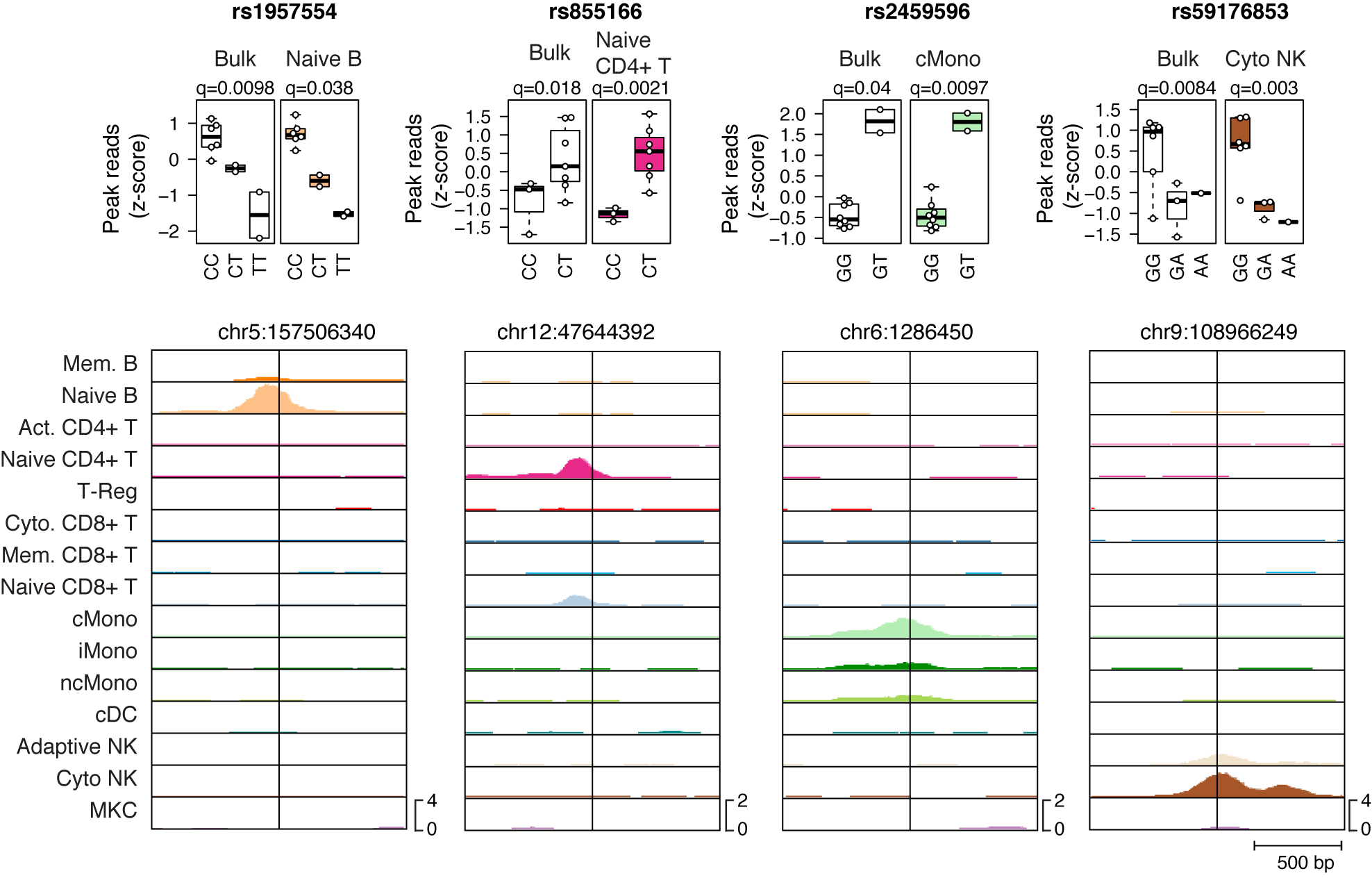

Supplement: S12 Fig — A) Examples of cell-type specific caQTLs due to presence of the peak in a single cell type. From left to right, rs1957554 is a caQTL for a naïve B cell-specific site, rs855166 is a caQTL for a naïve CD4+ T cell-specific site, rs2459596 is a caQTL for a classical monocyte-specific site, and rs59176853 is a caQTL for a cytotoxic NK cell-specific site. Top panels: color-coded boxplots show association in the different cell types, white boxplots show corresponding caQTL in “bulk” PBMCs. Association q-values are shown on the top and variant genomic location (hg19) is shown at the bottom. Bottom panels: genome-browser screenshot of snATAC-seq in different cell types. (TIF) [file pgen.1010759.s012.tif]

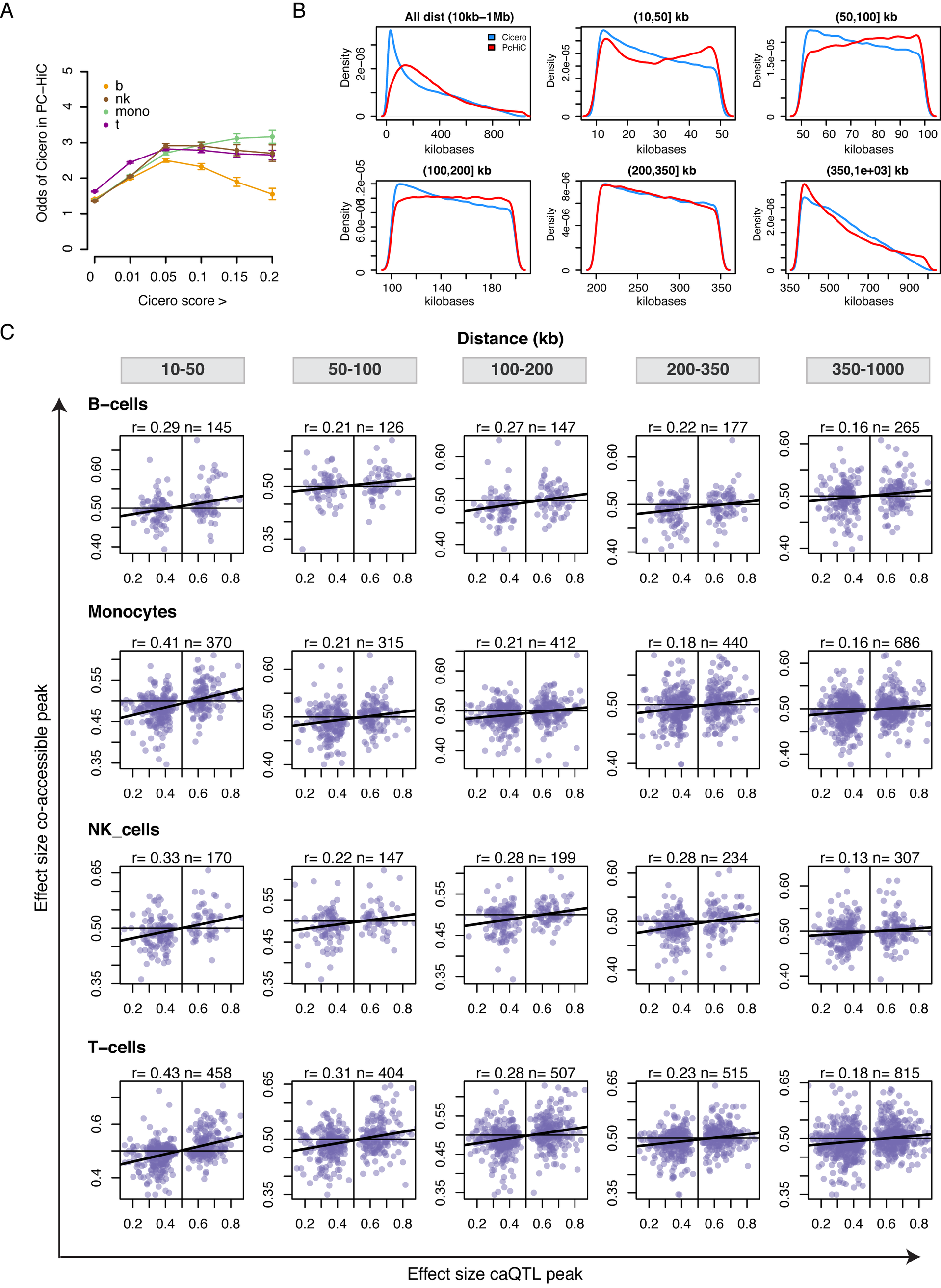

Supplement: S13 Fig — A) Enrichment of co-accessible sites in promoter-capture Hi-C loops at different cicero score thresholds. B) Comparison of distributions for promoter-capture Hi-C loops (red) or Cicero interaction (blue) across different distance bins. C) Correlation in the effects of caQTL variants on the QTL site and co-accessible promoter sites in each cell type, grouped by distance between the QTL site and co-accessible promoter site. Pearson correlation coefficient and number of co-accessible pairs of peaks are indicated. (TIF) [file pgen.1010759.s013.tif]

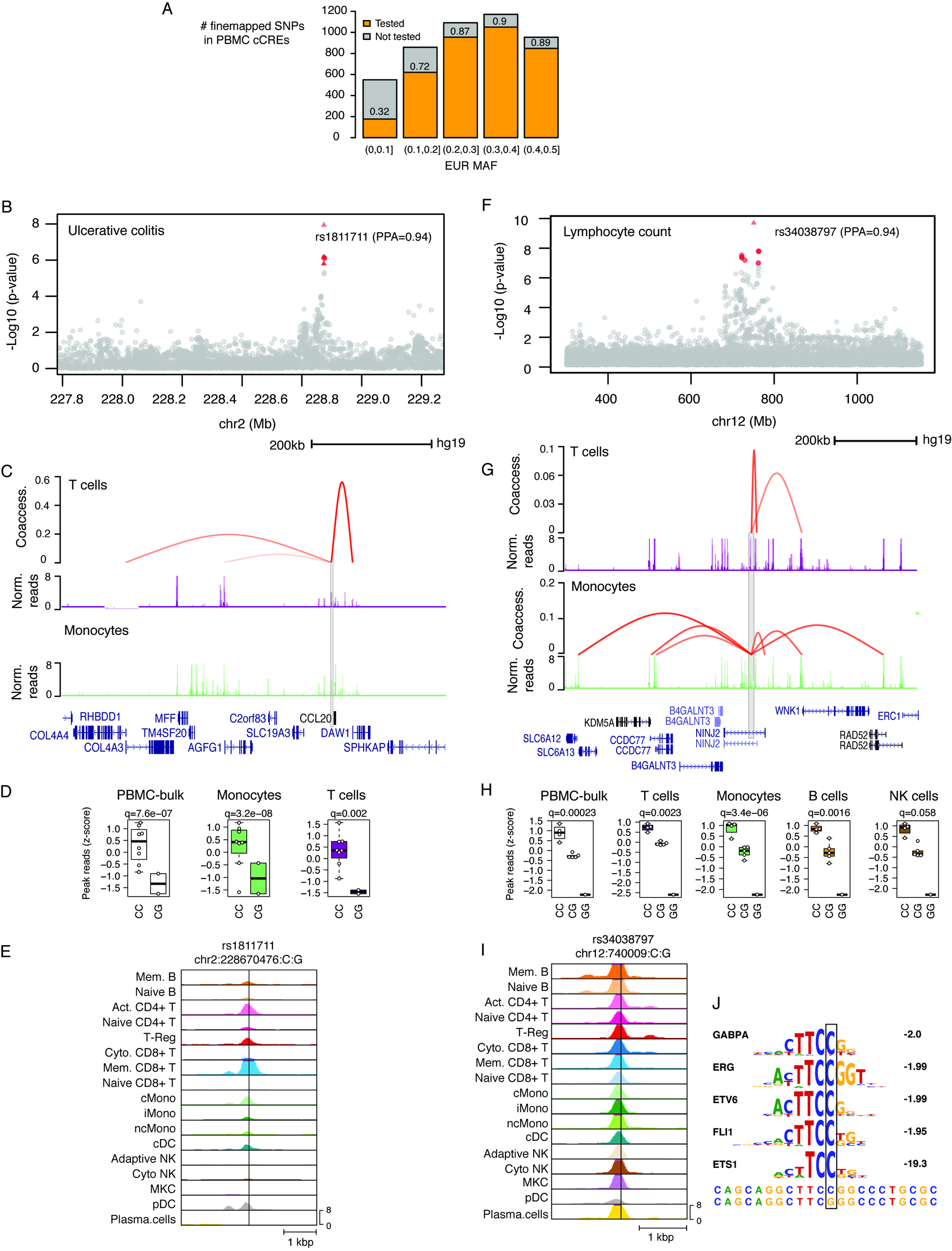

Supplement: S14 Fig — A) Minor allele frequency in 1000 Genomes EUR samples of fine-mapped variants tested for caQTL association in this study. B) Regional plot of the CCL20 locus on chr12 associated with ulcerative colitis, with credible set variants highlighted in red. Candidate causal variant rs1811711 is indicated with a triangle. C) Chromatin signal in T cells and Monocytes at the same locus and co-accessibility between the site harboring rs1811711 and DAW1, AGFG1, COL4A3 and COL4A4 promoters. D) Chromatin signal grouped by rs1811711 genotype in “bulk” PBMCs, Monocytes and T cells and q-values for caQTL association. E) Zoomed-in genome browser track of the caQTL peak in each sub-type. F) Regional plot of the locus on chr12 in the NINJ2 gene showing association with lymphocyte count, with credible set variants highlighted in red. Candidate causal variant rs34038797 is indicated with a triangle. G) Chromatin signal in T cells and Monocytes at the same locus and co-accessibility between the site harboring rs34038797 and CCDC77, WNK1, RAD52, NINJ2 and SLC6A12 promoter. H) Chromatin signal grouped by rs34038797 genotype in “bulk” PBMCs and all major cell types and q-values for caQTL association. I) Zoomed-in genome browser track of the caQTL peak in each sub-type. J) Predicted TF sequence motifs altered by rs34038797, where the variant base is highlighted. (TIF) [file pgen.1010759.s014.tif]
