# Supplementary material for: Mapping genetic effects on cell type-specific chromatin accessibility and annotating complex immune trait variants using single nucleus ATAC-seq in peripheral blood
Source: PLoS Genet. 2023 Jun 8;19(6):e1010759. doi: 10.1371/journal.pgen.1010759 (PMC10298776; doi:10.1371/journal.pgen.1010759)
Supplement: Attachment — Submitted filename: Comments.121222.docx [file pgen.1010759.s025.docx]

**We appreciate the positive comments from the editor and reviewers on our manuscript. Responses and corresponding changes in the manuscript are provided in-line with each comment.**

Reviewer #1: Benaglio & Newsome et al investigated non-coding GWAS variant function by generating snATAC-seq on sorted PBMCs from 13 individuals. Using this data, the authors identify chromatin accessibility QTLs (caQTLs), which they linked fine-mapped immune trait variants to putative effector genes using co-accessibility with promoters. The authors provide comprehensive validation of their dataset by demonstrating concordance to previously generated external datasets such as eQTLs and Promoter Capture HiC data in immune cell types as well as bulk caQTL from lymphoblastoid cell lines. The authors functionally validated the allelic effects for a likely causal variant, rs72928038, they provide a sufficiently detailed and thoughtful limitations section in their discussion, and they do not overinterpret their results.

The authors confirm that using snATAC-seq for caQTL mapping is a valuable approach to add to for resolving shared cell type specific differences in cis regulatory elements and provide insight into the mechanism of action for non-coding variant for multiple immune traits. These types of studies have been done in a number human tissues including immune cells, but the current study does represent added value to the field as these approaches mature and sample sizes increase.

Major Issues:

1. The functional follow up on the T1D rs72928038 variant and its target gene BACH2 is not novel. This functional association was made previously by Robertson et al. (Nature Genetics 2021). While this represents a valuable confirmation, this reduces the novelty of the study. Do this authors have functional validation of another SNP-gene pair they can highlight? Alternatively, can the authors explain how the way they validated this T1D pair is novel and/or value-added compared to the Robertson study?

**We thank the reviewer for this comment. In the Robertson et al study they demonstrated that rs72928038 at the *BACH2* locus was a chromatin QTL in sorted CD4+ T cells. We note that our study and findings were available as a preprint prior to the Robertson et al study being published, and they in fact cite our preprint in their manuscript.**

**There are several points of novelty in our findings relative to the Robertson et al study. First, the cCRE that rs72928038 affects was active in naïve CD4+ cells and not in other CD4+ T cell populations, whereas they profiled bulk CD4+ T cells. Second, the cCRE was also active in naïve CD8+ T cells which were not profiled in that study. Third, as we tested for caQTLs in other immune cell types, we could determine that the effects of rs72928038 appear specific to T cells relative to other immune cell types. Third, we determined that rs72928038 affects enhancer activity in T cells using gene reporter assays in addition to TF binding.**

**Therefore, our study builds on the published findings for rs72928038 in Robertson et al by showing that this variant affects a site specific to naïve CD4+ T cells relative to other CD4+ T cell populations as well as naïve CD8+ T cells, doesn’t appear to affect other immune cell types such as B cells, NK cells and monocytes, and affects enhancer activity in addition to transcription factor binding in T cells.**

Line 396: *“Our findings provide further insight into the specificity of cis-regulatory effects at the BACH2 locus across T cell sub-types and other immune cell types.”*

2. The uploaded main text figures have a resolution issue that makes several of their axes illegible.

**Thank you for highlighting this issue. We have confirmed that the figures included in the revised manuscript are high-resolution images.**

3. In Figure 3C, the trend for enrichment between Cicero predicted co-accessible regions and Promoter Capture HiC interactions appear to increase with distance. How does the distribution of distances between accessible regions and gene promoters in Promoter Capture HiC and Cicero coaccessibility? Are the distributions uniform across genomic distance or could some of the differences between the methods be explained by chromatin conformation/Cicero coaccessibility capturing interactions at different ranges?

**We thank the reviewer for highlighting this observation. We compared the distance between co-accessible links and pcHiC interactions across different size bins. At smaller size bins (e.g. 10-50kb, 50-100kb, 100-200kb) co-accessibility generally tends to identify relationships that cover shorter distances compared to pcHiC interactions, suggesting they may capture slightly different classes of cRE-target gene relationships. At greater distances (>200kb), the distributions are more closely matched, which may explain the stronger enrichment. We included this result as Supplementary Figure 13B and modified the main text and methods.**

Line 304: *“When segregating co-accessible sites by distance bins, we observed enrichment for pCHi-C loops across all bins with stronger enrichment at greater distances (Figure 3c, Supplementary Figure 13b).”*

4. Given that sample size is limiting to detect caQTL particularly in rare cell types, it would be useful to discuss how many more donors/cells are necessary for some of the rarer cells (Treg etc) to have comparable power to the more common cell types from this study. It may be useful to run a sampling simulation on a high frequency population (e.g., naive CD4+ T cells) to assess at what point reduced cell numbers hamper caQTL calling power.

**We thank the reviewer for this suggestion, which we agree would be a valuable addition to the manuscript.**

**To determine a minimum number of cells per sample required to call caQTLs,** **we down sampled the most common cell type, classical monocytes, to 10, 25, 50, 100, 250, 500, 1k, and 2k cells per individual, and re-performed caQTL mapping for each cell number. The number of detected caQTLs decreases as a function of the number of sub-sampled cells, and below 100 cells per sample almost no caQTLs are detected at FDR<.10. We next estimated the proportion of true associations (π1) among caQTLs detected in down-sampled cells, which was 0.88 for 100 cells, >0.97 for higher cell numbers, and 0 for lower cell numbers. Our conclusion from this analysis is that, assuming the same sequencing depth, at least 100 cells per sample is needed to generate caQTL statistics that are broadly concordant with those from much higher cell numbers, although the number of significant caQTLs is still low. We added these results to Supplementary Table 7 and describe the results in the main text and methods.**

Line 199: **“***To further determine the impact of cell number on caQTL discovery, we down-sampled cells from the most common cell type classical monocytes and re-performed caQTL mapping (see Methods). As expected, the number of detected caQTLs decreased as a function of the number of cells. Below 100 cells per sample, almost no caQTLs were detected at FDR<.10 and the proportion of caQTLs estimated to be shared (π1) with the original caQTLs decreased substantially (Supplementary Table 7).”*

Line 674: *“To estimate the sensitivity of caQTL detection across different cell numbers, we sampled 10, 25, 50, 100, 250, 500, 1k and 2k cells per individual from the cell population with highest cell count, classical monocytes (1,750.5 on average per sample, 10 samples). Barcodes were randomly sampled (with replacement in the case of 2k cells per individual) and used to subset classical monocyte BAM files for each sample. We then performed caQTL mapping using the exact same method as for regular cell sub-types. We determined sharing in each sub-sample with the original caQTLs by calculating the π1 statistic using the qvalue package in R^47^ as described by Chen et al^15^ with method =’bootstrap’.”*

5. While the authors addressed why the colocalization analyses were not performed in the limitations section of the discussion (lines 436-438), this section should be expanded to discuss any expected differences in interpreting the results between the current approach and colocalization.

**We agree with the reviewer that the conclusions one could draw from annotating GWAS variants by simple overlap with a caQTL versus formal colocalization analysis are different. We have clarified this point further in the discussion:**

Line 452: *“An additional caveat was that we only tested variants in peaks for association, as opposed to all variants in a window around each site, and were unable to formally compare caQTL and disease signals. Therefore, it is possible that a subset of complex trait signals overlapping with immune cell type caQTLs have a different causal variant and may not actually affect chromatin accessibility. Colocalization analyses will be needed to establish which signals have shared causal variants with caQTLs^34^.”*

Minor Issues:

6. I was confused by the labels of Sup Figure 9 (caQTL population +ASE and caQTL population only & population + ASE). The titles should either be renamed to clarify what is the difference between the groups or elaborated on in the figure legend.

**We apologize for the confusion, and we have now provided new labels and a better explanation in the figure legend.**

Line 1007: ***“****For each cell type (first 4 plots) and sub-type (remaining 15 plots), a scatter plot shows the consistency between caQTL effect (π) in the standard QTL analysis including allelic imbalance (x-axis,) and the effect for the same variant-peak pair excluding the allelic imbalance component (y-axis). The latter was obtained by running RASQUAL using the --population-only option. Black dots indicate caQTLs that were significant only in the standard analysis, and red dots indicate caQTLs that were significant in both analyses. The percentage of discordant effects are indicated.”*

7. Line 304: (Figure 3f is missing a closing parenthesis

**We have fixed this mistake.**

Reviewer #2: In this study by Benaglio et al. the authors perform single nucleus chromatin accessibility sequencing on a collection of PBMCs from 13 individuals. From a total of 96K nuclei profiled they identified 17 immune cell types and subtypes. They then performed allele-specific mapping of caQTLs across the different cell types to identify 6,901 caQTLs at 10% FDR. They further annotate putative target genes using co-accessibility analysis and performed fine-mapping of 16 immune traits. While this is an important study there are some limitations that could be addressed to improve the manuscript.

Specificcomments

1. The authors title should provide a more informative and specific title that mentions ‘circulating immune cells’ or PBMCs. Otherwise, it is not clear what cell/tissue was used for the mapping of the caQTLs.

**We thank the reviewer for the suggestion, and we have modified the title accordingly:**

Line 1**: *“****Mapping genetic effects on cell type-specific chromatin accessibility and annotating complex immune trait variants using single nucleus ATAC-seq in peripheral blood”*

2. In the introduction the authors claim that no other study has performed caQTL mapping in specific cell types using single cell assay. However, there was a recent study published in Nature Genetics by Turner A et al. (PMID 35590109) that mapped cell type specific caQTL using snATAC in coronary artery. The authors should revise the sentence to acknowledge this recent study and cite the paper.

**We apologize for this oversight. At the time of the original submission and preprint our study was the first to perform caQTLs using single cell data, and we mistakenly did not add a citation for the more recent work from Turner *et al* in this current submission. We modified the introduction to include this reference.**

Line 90: ***“****Several recent studies have mapped QTLs using cell type-specific profiles derived from single cell assays^18–20^.”*

3. The authors obtained an impressive number of cells per sample however there should be more details on how there were more numbers from the “Lab 10X pipeline” when the CellRanger estimates for ATAC often inflates the number based on debris on “cell-like” counts. Also more details on the filtering criteria such as minimum number of fragments per cell and TSS enrichment score cut-off would be useful.

**We apologize for the lack of clarity in these descriptions. The column of Table S2 with the number of cells after ‘Lab 10x pipeline’ (in the Github repository for this manuscript) refers to the initial ‘pre-filtering’ step which removes cell barcodes with less than 4,000 mapped reads. There were on average 9.1k cells per sample after this pre-filtering. Additional filtering of cells was then done across multiple rounds of clustering and sub-clustering, where clusters or sub-clusters of cells with low quality metrics such as FRiP, usable reads, fraction of reads in promoter and marker gene profiles indicative of doublets and other artefacts were removed. These additional filters led to a ~20% reduction in cell numbers from the pre-filtering step, where on average there were 7.4k cells per sample.**

**We modified the header in Supplementary Table 2 to describe more explicitly the number of cells after the ‘pre-filtering’ step. We also added the final number of cells per sample after all filtering steps as a new column in Supplementary Table 2. Both the pre-filtering step as well as the additional filtering steps are described in more detail in the methods:**

Line 516: *“For each sample, we retained nuclei with a minimum read count of 4,000. After this initial pre-filtering step there were on average 9.1k nuclei per sample (Supplementary Table 2). Individual sample matrices were then concatenated using python AnnData package (117,824 total nuclei). Using scanpy^38^ (v.1.5), we extracted highly variable windows using mean read depth and normalized dispersion (min_mean=0.01, min_disp=0.25). After normalization to uniform read depth and log-transformation, we regressed out the log-transformed read depth within highly variable windows for each cell. The “pca” function in scanpy was then used to perform principal component analysis. The top 50 principal components were extracted and corrected to remove batch effects using harmony^39^ version 0.1.0 (arguments “theta=c(2,2) and “do_pca=FALSE”), using sample donor ID as a covariate. The harmony-corrected components were then used to calculate the nearest 30 neighbors using the cosine metric. This cosine metric was then used to perform UMAP dimensionality reduction clustering with the parameters ‘min_dist=0.3’, and Leiden clustering^40^ (resolution=1.5). To filter nuclei with low quality, entire clusters with low fraction of reads in promoters, low log usable read count, low fraction of reads in peaks, and/or clusters that did not have uniform representation across samples were iteratively removed across multiple rounds of clustering. A total of 15,958 nuclei were removed by this approach. We then performed sub-clustering of each cluster using the Louvain algorithm (resolution=2) and removed sub-clusters within each cluster with higher read counts or accessibility for multiple cell type marker genes (n=5,864 nuclei removed). At the final round of clustering, we removed the nuclei from 10x public datasets at just retained the nuclei in the 13 PBMC samples in our study. After all filtering steps and final clustering with 13 PBMC samples, the final map consisted of 96,002 nuclei mapping to 17 clusters. There were on average 7.4k nuclei per sample in the final map, a 20% reduction from the starting number of pre-filtered nuclei (Supplementary Table 1).”*

4. The authors provide key cell-specific literature references for the cell type annotation. Have they also considered using an automated immune cell reference such as CellTypist to validate some of these?

**We appreciate the reviewer comment and agree that automated methods are increasingly valuable tools for cell annotation. Existing methods for automated cell annotation such as CellTypist and Azimuth, as far as we are aware, currently use references derived from single cell RNA-seq, and existing models that they use have been trained on and optimized for gene expression data. Over time as models are developed and trained on high-resolution datasets with accessible chromatin as a modality, we agree these would help validate cell type labels and this point was included in the discussion:**

Line 461: ***“****A further limitation is that, due to the sparsity of single cell profiles and current methods for data processing and clustering, cell type clusters will often contain misclassified and doublet cells, although this appears to have limited impact on caQTL mapping in our study. Methods such as automated labeling or single cell multimodal assays will help more accurately resolve cells^35^.”*

5. The number of caQTLs by cell type is high despite by the smaller number of individuals profiled. The mention of 6,901 total caQTLs is based on FDR of 10%, which is quite lenient. The authors should report the number of caQTLs mapped at a more commonly used threshold (5%) in the main text and abstract

**We have amended the abstract and main text to report the number of caQTLs detected at an FDR threshold of 5%.**

Line 43: “*We mapped chromatin accessibility QTLs (caQTLs) in each immune cell type and sub-type using individuals of European ancestry which identified 6,901 caQTLs at FDR<.10 and 4,220 caQTLs at FDR<.05*”

Line 163: *“A consistent proportion of caQTLs reached more stringent FDR thresholds (Supplementary Figure 6a). For example, at FDR<.05 we identified 4,220 caQTLs, including 3,162 at cell type resolution and 3,654 at sub-type resolution.”*

6. The authors should calculate the pi1 statistic (if not already included) to report the number shared true positive between the individual cell type caQTL and the merged bulk caQTL analysis. The authors performed rigorous sensitivity analyses to determine the drivers of the caQTL discoveries, however this additional support would be helpful

**We thank the reviewer for this suggestion. We calculated the π1 statistic for caQTL sharing described in Chen et al. Cell, 2016. The proportion of caQTLs (π1) shared with ‘bulk’ caQTLs was high across cell types (mean π1=0.87, median π1=0.96). One notable exception is megakaryocytes, which has very low cell counts and a very different chromatin profile relative to other immune cell types (see UMAP plot in Figure 1), and therefore it may not be surprising that there is low sharing with ‘bulk’ caQTLs for this cell type.**

**This analysis is now included in Supplementary Table 7, and described in the main text and methods**

Line 213: *“Finally, caQTLs in each cell type and sub-type showed a high degree of sharing with “bulk” caQTLs (median π1=0.96) (Supplementary Table 7).”*

Line 679: *“We determined sharing in each sub-sample with the original caQTLs by calculating the p1 statistic using the qvalue package in R^47^ as described by Chen et al^15^ with method =”bootstrap”. We also calculated sharing between cell type and sub-type caQTLs and ‘bulk’ caQTLs using the same p1 statistic.”*

7. The motif enrichment revealed both shared and cell type specific TF motifs. Were the cell type specific enriched motifs correlated with higher differential gene expression for those TF genes? Some further discussion on the redundancy of the TF families as well as cell type specific TFs underlying functionally distinct immune cell types and subtypes for different traits would be helpful.

**We appreciate the reviewer comment. In our study we do not have matched scRNA-seq data, so we instead used a published scRNA-seq dataset from PBMCs. For TF sequence motifs enriched among cell-type caQTLs, we identified TF genes preferentially expressed in the same cell type from scRNA-seq. There were numerous examples of TF genes with cell type-specific expression patterns that match motif enrichments in cell type-specific caQTLs, and these results are included in Supplementary Table 8. Several examples are in highlighted Figure 2G including SPIB in B cells and RUNX3 for NK and T cells. We have added these results in Supplementary Table 8 and modified the relevant sections of results and methods**

Line 249: *“Motifs disrupted by caQTLs included those with broadly shared enrichment across different cell types such as ETS family motifs, as well as those with cell type-specific enrichment such as FOS/JUN and CEBP motifs in monocytes, and RUNX motifs in NK and T cells. As TFs in the same structural sub-family often share similar binding motifs, we next determined the expression of TF genes using published PBMC scRNA-seq. We identified TF genes with cell type-specific expression (FDR<.05, LogFC>1; Wilcoxon test) that matched motif sub-family enrichment patterns (Supplementary Table 8), implicating specific TFs likely altered by immune cell type caQTLs.”*

Line 736: *“To determine which TF genes may underlie sequence motifs enriched for caQTLs in each cell type, we used a published scRNA dataset in PBMCs^21^. For each of the 4 major cell types (monocyte, T cell, B cell, NK cell) in scRNA-seq data, we applied the ‘rank_gene_groups’ function in scanpy with the arguments ‘method=wilcoxon’ and ‘corr_method= benjamini-hochberg’. We identified TF genes preferentially expressed in each cell type cluster with respect other clusters with FDR<0.05 and log fold change >1, and matched TFs genes to corresponding TF sequence motifs”*

**We also modified the discussion to highlight that TFs in the same structural sub-family often share similar sequence motifs, which makes determining the specific TFs driving cell type-specific regulation difficult from accessible chromatin data alone.**

Line 442: ***“****Furthermore, we identified candidate transcriptional regulators through which genetic effects on chromatin accessibility in each cell type are mediated, although as TFs in the same structural sub-family often share similar motifs the specific TFs can be difficult to determine from accessible chromatin alone.”*

8. The authors should consider integration of their PBMC snATAC-seq data with PBMC snRNA-seq data to determine whether some of the cell type specific accessible regions correlate with gene expression.

**We appreciate the reviewer suggestion and agree that correlative analyses of snATAC-seq and snRNA-seq can reveal relationships between accessible chromatin and gene expression. Given the increasing availability of assays which generate paired profiles of accessible chromatin and gene expression from the same cell, in our view correlative analyses to link chromatin and expression profiles will be best performed from these datasets as opposed to integrated single modality data. We have modified the discussion to highlight this point:**

Line 465: ***“****Data from multimodal assays will also be valuable in defining correlative relationships between accessible chromatin and gene expression.”*

9. In Supp Fig 12, the box plots showing allelic effects on chromatin accessibility at rs9394346 in the bulk sample look statistically significant, compared to the cell type specific box plot for Cyto NK. Also, the box plots for a few other cell types appear to be significant however they have high q-values. The authors should explain whether these peak reads are mapping to a different variant, or this is a limitation of RASQUAL.

**We thank the reviewer for highlighting this point. The boxplots just show normalized read counts and do not incorporate allelic imbalance, which is a major driver of caQTL discovery in our study, nor do they account for covariates included in the model when calculating caQTL statistics. Therefore, a limitation of these boxplots is that there is the potential for a visual inconsistency between the normalized read counts and the results of caQTL mapping which consider additional information.**

**For rs9394346 specifically, we confirm that the same variant was shown in the plots for each cell type and bulk. After inspection of the reads mapping to the variant, the discrepancy is likely because when mapping caQTLs in each cell type duplicate reads are marked at the cell level – as each cell is its own library – whereas in ‘bulk’ data duplicate reads are marked at the sample level to mirror a true ‘bulk’ experiment more closely. Therefore, there are fewer reads available for allelic imbalance mapping in ‘bulk’ and therefore this variant is not significant in the bulk analysis. This point is highlighted in the discussion of the manuscript on Line 429.**

**As the boxplots do not visually capture the allelic imbalance effect driving differences in significance for this variant, we decided to remove it from the manuscript to avoid confusion.**

10. It might be more helpful to provide cell-type specific bed files in addition to the Supp Table 5.

**We have uploaded the bed files for peak calls in individual cell types to Zenodo together with all the supporting data for this study.**

Line 866: ***“****All processed data underlying analyses and figures in the paper are located at https://doi.org/10.5281/zenodo.7375095 and in the supplementary tables where specified.”*

Have all data underlying the figures and results presented in the manuscript been provided?
Large-scale datasets should be made available via a public repository as described in the *PLOS Genetics* data availability policy, and numerical data that underlies graphs or summary statistics should be provided in spreadsheet form as supporting information.

Reviewer #1: Yes

Reviewer #2: **No:** It appears that data from only 7 of the 13 total individual PBMC samples have been uploaded to GEO.

**The other 6 samples were included in a previous publication and are available at another accession number (GSE163160) – they are also referenced in GSE199253 directly under a ‘re-analysis’ header. To avoid any confusion, however, we have added the GSE163160 accession number to the data availability section.**

Line 864: *“Raw sequence data are available in NCBI GEO accession GSE199253 and GSE163160.”*
